# Long-Term Expansion of Porcine Intestinal Organoids Serves as an *in vitro* Model for Swine Enteric Coronavirus Infection

**DOI:** 10.3389/fmicb.2022.865336

**Published:** 2022-03-14

**Authors:** Min Zhang, Lilei Lv, Hongming Cai, Yanhua Li, Fei Gao, Lingxue Yu, Yifeng Jiang, Wu Tong, Liwei Li, Guoxin Li, Guangzhi Tong, Changlong Liu

**Affiliations:** ^1^College of Veterinary Medicine, Northeast Agricultural University, Harbin, China; ^2^Shanghai Veterinary Research Institute, Chinese Academy of Agricultural Sciences, Shanghai, China; ^3^Key Laboratory of Fujian-Taiwan Animal Pathogen Biology, College of Animal Sciences, Fujian Agriculture and Forestry University, Fuzhou, China; ^4^College of Veterinary Medicine, Yangzhou University, Yangzhou, China; ^5^Jiangsu Co-innovation Center for the Prevention and Control of Important Animal Infectious Disease and Zoonosis, Yangzhou University, Yangzhou, China

**Keywords:** long-term 3D culture, porcine intestinal organoids, swine enteric coronaviruses, interferon-stimulated genes, immune suppression

## Abstract

A reliable and reproducible model *in vitro* for swine enteric coronaviruses infection would be intestinal models that support virus replication and can be long-term cultured and manipulated experimentally. Here, we designed a robust long-term culture system for porcine intestinal organoids from the intestinal crypt or single LGR5^+^ stem cell by combining previously defined insights into the growth requirements of the intestinal epithelium of humans. We showed that long-term cultured swine intestinal organoids were expanded *in vitro* for more than 6 months and maintained the potential to differentiate into different types of cells. These organoids were successfully infected with porcine enteric coronavirus, including porcine epidemic diarrhea virus (PEDV) and transmissible gastroenteritis virus (TGEV), and were capable of supporting virus replication and progeny release. RNA-seq analysis showed robust induction of transcripts associated with antiviral signaling in response to enteric coronavirus infection, including hundreds of interferon-stimulated genes and cytokines. Moreover, gene set enrichment analysis indicated that PEDV infection could suppress the immune response in organoids. This 3D intestinal organoid model offers a long-term, renewable resource for investigating porcine intestinal infections with various pathogens.

## Introduction

Porcine epidemic diarrhea virus (PEDV), transmissible gastroenteritis virus (TGEV), and porcine delta coronavirus (PDCoV) are major pathogenic swine enteric viruses, which belong to the family Coronaviridae. PEDV, TGEV, and PDCoV are highly contagious and are a major cause of illness and death in piglets (Jung and Saif, 2015; Jung et al., 2016). These viruses can cause endemics or large-scale epidemics in major pig-producing countries, which lead to severe economic losses in the swine industry. So far, no effective treatments are available for any of these swine enteric coronaviruses (CoVs) that have resisted eradication efforts in different countries (Vlasova et al., 2020).

Swine enteric CoVs share tropism for swine intestinal epithelial cells *in vivo* (Sungsuwan et al., 2020). Infection of any of these enteric coronaviruses can damage the jejunum and ileum of neonatal piglets up to 3 weeks of age (Vlasova et al., 2020). However, most *in vitro* studies of swine enteric CoVs have been performed in non-targeting cell lines. Vero cells from African green monkey kidneys have been used for PEDV propagation since it was described by Hofmann and Wyler (1988). Other immortalized cell lines permissive for PEDV include MARC-145, LLC-PK1, Huh7, and so forth (Liu et al., 2015; Zhang et al., 2016, 2017). PK-15 cells derived from porcine kidney and swine testicle-derived ST cells are used for TGEV isolation and propagation *in vitro* (Wang et al., 2019). The conclusions obtained from those non-targeting cells may not represent the true response to virus infection in targeting cells. Therefore, several swine intestine epithelial cell lines were developed like IPEC-J2 and IPI-21, which derived from pig jejunum and ileum, respectively. However, the viral infectivity in IPEC-J2 and IPI-21 cells are controversial, and it is increasingly evident that these cell models are not sufficient to fully understand swine enteric CoVs diseases (Zhao et al., 2014; Wang et al., 2019). Thus, the development of a reliable and reproducible model *in vitro* for swine enteric Covs infection is urgently needed for investigations of CoVs.

Unlike the immortalized cell lines models, organoids are self-organizing 3D structures grown from adult stem cells and recapitulate key aspects of the organ from which those cells derive (Clevers, 2016). The establishment of mouse intestinal organoids in 2009 advanced our understanding of the intestinal epithelium and led to numerous opportunities to study virus pathogenesis and host-pathogen interactions in this particular model (Sato et al., 2009; Artegiani and Clevers, 2018). Long-term cultures in which organoids can differentiate and recapitulate normal crypt-villus architecture have been established from the mouse and human intestines (Sato et al., 2011). Organoids derived from the porcine intestine have been produced using different culture systems (Powell and Behnke, 2017; Derricott et al., 2019; Li et al., 2019; Li Y. et al., 2020; Vermeire et al., 2021). However, these approaches may not allow long-term expansion of swine intestinal epithelium from adult pig individuals *in vitro*.

Long-term expansion of organoids culture requires suitable matrices creating a 3D condition as well as the appropriate medium providing necessary niche factors, which recapitulates the *in vivo* stem cell niche and able to sustain stem cell the capacity for self-renewal and the potential to differentiation (Clevers, 2016; Artegiani and Clevers, 2018). The media supplemented with wnt3a, R-spondin1, noggin, and the epidermal growth factor is sufficient to support the long-term expansion of mouse intestinal organoids, whereas the addition of nicotinamide, along with an inhibitor of ALK and an inhibitor of p38, was required for long-term culture of human small intestine organoids (Sato et al., 2011).

In this study, we designed a long-term culture to robust expand the porcine intestinal organoids by combining previously defined insights into the growth requirements of the intestinal epithelium. Studies of these cultures indicated that those swine intestinal organoids could self-renew for 6 months. These intestinal organoids were susceptible to infection by porcine enteric coronavirus PEDV and TGEV. We conclude that long-term expansion organoids represent versatile models for the *in vitro* study of swine enteric coronaviruses infection.

## Materials and Methods

### Animal and Viruses

All experimental procedures and animal care protocols were approved by the Care and Use of Laboratory Animals of Shanghai Veterinary Research Institute (SHVRI), Chinese Academy of Agricultural Sciences, China. The PEDV-SD strain (GenBank accession No.MZ596343) and TGEV H16 (GenBank accession no.FJ755618) stocks were prepared and titrated on the Vero E6 cells and LLC-PK1 cells by TCID50, respectively.

### Cell Culture and Conditioned Media

L-WRN cells (CRL-3276) were obtained from American Type Culture Collection, and cultured in high glucose DMEM (Hyclone) supplemented with penicillin (100 U/mL), streptomycin (100 μg/mL) (Sigma-Aldrich), Geneticin (500 μg/ml) (Invitrogen), Hygromycin (500 μg/ml) (Invitrogen; Cat#: 10687010), and 10% fetal bovine serum (Gibco). Mycoplasma infection screening was performed with the LookOut Mycoplasma PCR Detection Kit (Sigma-Aldrich) as recommended by the manufacturer using cultured cell supernatant. The L-WRN conditioned media was made as previously described using the L-WRN cell line (Miyoshi and Stappenbeck, 2013).

### Porcine Intestinal Tissue Processing, Crypt Isolation, and Organoids Culture

Porcine intestinal crypts from the duodenum, jejunum, or ileum were prepared from specific-pathogen-free 10 days old piglets using previously described protocols with minor modification (Fujii et al., 2015). Briefly, the intestinal sections were cut and opened longitudinally. The mucosal layer and villi were removed by scraping with a microscope slide, and 2-4 cm of intestinal sections were cut into 5-mm segments. The dissected pieces were washed 5-10 times with PBS until the supernatant was free of debris, and then the washed pieces were incubated in cold PBS supplemented with 2.5 mM EDTA for 40 min at 4°C with gently rotating. Then pieces were pelleted and pipetted up and down in 10 ml of cold PBS vigorously to release crypts. The supernatant containing crypts was centrifuged at 400 *g* for 3 min. The crypts were washed twice with cold PBS and resuspended in Matrigel (Corning) with about 100–150 crypts per 50 μl of Matrigel. Dispense 50 μl of the crypt-Matrigel suspension into the center of each well of a 37°C pre-warmed 24-well plate. The Matrigel was polymerized for 15 min at 37°C, porcine intestinal organoids (PIO) medium plus 10 μM of ROCK inhibitor was added, and crypts were incubated at 37°C, 5% CO_2_. The composition of PIO medium is: Advanced DMEM/F12, 100 μg/ml primocin, 2 mM GlutaMax, 10 mM HEPES, 1 × N2, 1 × B27, 1 mM N-Acetyl-Cysteine, 10 mM Nicotinamide, 50% L-WRN conditioned medium, 50 ng/ml human EGF, 500 nM A83-01 and 3 μM SB202190 (Supplementary Table 1).

### Organoids Passage, Cryopreservation, and Resuscitation

The intestinal organoids culture medium was refreshed every 3 days until organoids were approximately ten times the original size. Matrigel was broken up with medium using a 1-ml pipet tip and transferred from the well to a 15-ml tube to passage the organoids. The organoids were centrifuged at 400 *g* for 3 min, and the medium and most of Matrigel were removed. The organoids were incubated with 1 ml of TrypLE Express (Invitrogen) at 37°C for 5 min. Basal medium (Advanced DMEM/F12, 100 U/ml penicillin, 100 μg/ml streptomycin, 2 mM GlutaMax, 10 mM HEPES) was added, and cells were spun down at 400 g for 3 min. The pellet was resuspended in Matrigel and plated in droplets of 50 μl each well of 24-well plate. After allowing the Matrigel to solidify, PIO medium supplemented with 10 μM ROCK inhibitor was added to the plates, and organoids were cultured at 37°C 5% CO_2_.

On day 4 or 5 of culture, the medium was removed from wells, and 1 ml of Recovery Cell Culture Freezing Medium (Invitrogen) was added. Organoids-containing Matrigel was scraped and transferred into 2-ml cryovials. Place tubes in a freezing container and incubate the tubes at −80°C for at least 1 day. Then transfer the frozen cryovials to a liquid nitrogen storage tank.

To recover the frozen organoids, cryovials containing organoids were removed from liquid nitrogen and thawed quickly in a 37°C water bath. The organoids were transferred into a 15-ml centrifuge tube, and 10 ml of basal medium were added. The organoids were pelleted down at 4°C at 400 g for 3 min. Then, the organoids were resuspended with Matrigel and plated in droplets of 50 μl each well of 24-well plate. After allowing the Matrigel to solidify, PIO medium supplemented with 10 μM ROCK inhibitor was added to the plates, and organoids were cultured at 37°C, 5% CO_2_.

### Organoids Differentiation

For swine intestinal organoid differentiation, organoids were plated in Matrigel with PIO media. At 2–3 days after plating in Matrigel, organoids were cultured with PIO medium with DMSO or PIO medium 5% L-WRN conditioned medium containing 5 mM DAPT for 2 days. After treatment, organoids were harvested, and the total RNAs were purified using Trizol.

### Organoids Proliferation Assay and 5-Ethynyl-2′-Deoxyuridine Imaging

Single-cell suspensions from swine intestinal organoids were generated by TrypLE and counted. About 3,000 cells per replicate were plated in Matrigel and incubated in the PIO medium described above. At the indicated time points, 700 uM resazurin (Sigma) in PBS was added 10% by volume to each treatment well. Cells were incubated for 5 h in 37°C, 5% CO_2_ incubator, and then fluorescence was measured on BioTek Epoch Microplate Spectrophotometer (BioTek).

The proliferation of organoids was also evaluated by Click-iT Plus EdU Imaging Kit (Invitrogen). In brief, organoid cells were incubated with EdU for 6 h fixed in 4% paraformaldehyde in DPBS and incubated for 15 min at room temperature. After washing 3 times with DPBS, the organoids were permeabilized and blocked with blocking buffer (0.1% Triton-X 100 and 3% BSA in DPBS) for 20 min at room temperature. Click-iT Plus reaction cocktail was added and incubated with cells for 30 min. The cells were counterstained with Hoechst for 10 min. Images were taken by Olympus IX81 fluorescence microscope.

### Histology and Imaging

Organoids were collected and fixed in 4% paraformaldehyde (PFA). Pellet fixed organoids at 400 g for 5 min, and wash with 1 ml PBS. Aspirate supernatant as much as possible, then mix 20 μl of organoids at 1:1 with molten 2% low melt agarose to tube. Immediately pipette onto parafilm to form a small dome, allow to cool. Then the small dome was put in a mold and filled with low melt agarose to form a block, followed by dehydration, paraffin embedding, sectioning, and standard H&E staining. Images were acquired on a Leica Eclipse E600 microscope.

### Quantitative Reverse Transcription Polymerase Chain Reaction

Total RNA was purified from organoids using TRIzol (Thermo Fisher Scientific, Shanghai, China). The reverse transcription reaction was carried out with 1 μg of total RNA using a PrimeScript first-strand cDNA synthesis kit (Takara, Beijing, China). Quantitative real-time PCR was performed using SYBR Premix Ex Taq (Takara, Beijing, China) and a LightCycler 96 instrument (Roche, Shanghai, China). The relative quantities of the genes were calculated using GAPDH as a reference, using the formula: 2^–[*Ct(Gene)–Ct(GAPDH)*]^. The primer sequences for each gene are provided in Supplementary Table 2.

### Transmissible Gastroenteritis Virus Infection and Porcine Epidemic Diarrhea Virus

Organoids were harvested in a cold basal medium washed once to remove Matrigel and digested using TrypLE express. After digestion, organoids were washed once in a Basal medium. TGEV and PEDV infections were performed at a multiplicity of infection (MOI) of 0.1 in Basal medium (Trypsin was added to Basal medium at a concentration of 5 μg/ml for PEDV infection). After 2 h of virus adsorption at 37°C 5% CO_2_, cultures were washed twice with excess Basal medium to remove unbound virus. Organoids were re-embedded into 50 μl of Matrigel in 24-well tissue culture plates and cultured in 500 μl of PIO medium. Each well contained about 100,000 cells. Samples were taken at indicated time points by harvesting the medium in the well (supernatant) and the cells by resuspending the matrigel droplet containing organoids into 500 μl Basal medium.

### Immunofluorescent Staining of Organoids

Remove the culture medium from the wells, add 1 ml of ice-cold cell recovery solution (Corning) to each well and collect all organoids to a 15-ml tube. Fill up to 10 ml with ice-cold 1% (wt/vol) PBS-BSA and spin down at 300 g for 5 min at 4°C. Remove the supernatant. Gently resuspend the pellet of organoids in 1 ml of 4% PFA and incubate at 4°C for 45 min. Wash the pellet of organoids twice with 10 ml cold PBST and spin down at 300 g for 5 min at 4°C. For blocking the organoids, resuspend the pellet in 1 ml cold blocking buffer (0.1% triton-X100, 1% BSA in PBS). Incubate at 4°C for 15 min and wash the organoids’ pellet twice with 10 ml ice-cold 1% BSA/PBS and spin down at 300 g for 5 min at 4°C. Resuspend the pellet of organoids in mouse monoclonal antibody against TGEV N (1:800 Shanghai Youlong Biotech) or rabbit monoclonal antibody against PEDV N (1:500, Shanghai Ango Biotech) and incubate at 4°C overnight. Wash the pellet of organoids twice with 10 ml ice-cold PBS and spin down at 300 g for 5 min at 4°C. Resuspend the pellet of organoids in 1 ml of goat anti-mouse IgG (H + L) Cross-Adsorbed Secondary Antibody, Alexa Fluor 488 (1:1000, Invitrogen) or goat anti-rabbit IgG (H + L) Cross-Adsorbed Secondary Antibody, Alexa Fluor 488 (1:1000, Invitrogen) and incubate at 37°C for 1 h. Wash the pellet of organoids twice with 10 ml ice-cold PBS and spin down at 300 g for 5 min at 4°C. Resuspend the pellet of organoids in 1 ml 0.1% DAPI in PBS incubate at room temperature 15 min. Wash the pellet of organoids twice with 10 ml ice-cold PBS and spin down at 300 g for 5 min at 4°C. Add 50 μl of fructose-glycerol clearing solution (mix 33 ml of glycerol, 7 ml of dH2O, and 29.7 g of fructose) using a 200-μl tip with the end cut off and resuspend organoids gently. Cut off the end of a 200-μl tip and use it to transfer 20 μl of the organoids in the middle of the slide and place a coverslip on top. Gently apply pressure to both sides of the coverslip to firmly attach. Images were taken by Nikon Model Eclipse fluorescence microscope.

### RT-qPCR Assay for Viral Genome Copy Number

For viral genome copy number detection, total RNA was extracted from cultured organoids with RNeasy Mini Kit (QIAGEN), and RNA in the supernatant was extracted with QIAamp Viral RNA Mini Kit. RNA was then reverse transcribed into cDNA by reverse transcriptase mix (Takara). Total RNAs were extracted and then was synthesized into cDNA by reverse transcriptase (Thermo Fisher Scientific). Quantitative real-time PCR experiments were performed in triplicate to detect the sequence that codes nucleocapsid of PEDV or TGEV. Absolute quantitative RNA levels were calculated using standard curves derived from a virus stock.Primers used in this experiment: TGEV-qPCR-F: 5′-CTTCAACCCCATAACCCTCCAG-3′; TG EV-qPCR-R: 5′-GCCCTTCACCATGCGATAGC-3′; PEDV-qP CR-F: 5′-GAAGGCGCAAAGACTGAACC-3′; PEDV-qPCR-R:5′-TTGCCATTGCCACGACTCCT-3′.

### RNA-Seq and Analysis

Total RNA was extracted from porcine intestinal organoids using the miRNeasy Minikit (Qiagen). RNA quality was assessed by an Agilent bioanalyzer, and 1 μg was used for library preparation using the TruSeq Stranded mRNA Library Preparation kit (Illumina) per the manufacturer’s instructions. Sequencing was performed on an Illumina Novaseq 6000. RNA-seq FASTQ data were processed and mapped to the sus scrofa reference genome (sus scrofa11) using TopHat. Uniquely mapped reads were assembled into genes guided by Ensembl sus scrofa annotation file^1^. Differentially expressed genes between infections were evaluated using DESeq2 (Love et al., 2014) at a significance cutoff of FDR (False Discovery Rate) *P* < 0.01 unless otherwise stated. Pearson’s coefficient was calculated using the cor function with default parameters in R^2^. The hierarchical clustering analysis of samples and gene expression patterns in different samples was carried out using the ComplexHeatmap function (Gu et al., 2016). Heat maps based on z-score of FPKM values were also generated using ComplexHeatmap. Gene set enrichment analysis (GSEA) (Subramanian et al., 2005) was performed with GSEA 4.1.0 software^3^. Briefly, All log2FoldChange of annotated genes from DESeq2 output were ranked and the analysis was performed using the standard weighted enrichment statistic against gene sets derived from the GO (Gene Ontology) Biological Process ontology in the Molecular Signatures Database^4^. All RNA-seq data sets have been deposited in the Gene Expression Omnibus database with the accession number GSE182240.

### Statistical Analysis

All data were analyzed with R (see text footnote 2)and GraphPad Prism 9 (GraphPad, San Diego, CA, United States) and provided as mean ± SD unless otherwise indicated. Statistical analyses were performed using an unpaired Student’s *t*-test. The significance level (*P* value) was set at < 0.05 (*), < 0.01 (^**^), and < 0.001 (^***^).

### Data Availability Statement

All RNA-seq data sets have been deposited in the Gene Expression Omnibus database with the accession number GSE182240. All other data is available from the author upon reasonable request.

## Results

### Generation and Long-Term Expansion of Epithelial Organoids From Porcine Duodenum, Jejunum, and Ileum

A reliable and reproducible model *in vitro* for swine enteric coronavirus infection would be intestinal models that support virus replication and can be cultured long-term. Organoids derived from the porcine intestine have been produced, whereas long-term expansion of swine intestinal organoids has not been well established (Derricott et al., 2019; Li et al., 2019; Li Y. et al., 2020). To explore the long-term culture for porcine small intestinal organoids, we apply the culture medium of human intestinal organoids with minor modifications to the culture of porcine intestinal organoids.

**FIGURE 1:**
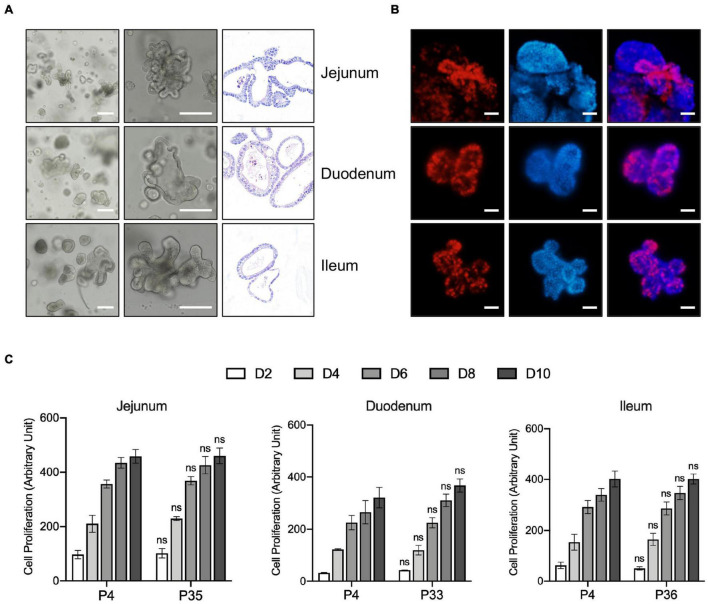
Generation, Characterization, and long-term expansion of porcine intestinal organoids. **(A)** Left and middle panel: representative images show the morphology of intestinal organoids from early passage of the porcine duodenum, jejunum, and ileum. Right panel: Representative H&E staining images of the porcine intestinal organoid showing the structure of organoids. Scale bar = 500 μm. **(B)** Organoids were cultured for 4 days and incubated with the thymidine analog EdU (red) for 6 h. Images were analyzed by fluorescence microscopy, and nuclei were counterstained with Hoechst (blue). Scale bar = 200 μm. **(C)** The proliferation capacity of porcine duodenum, jejunum, and ileum organoids at early and late passage numbers was measured by resazurin at the indicated time points. Statistics analysis was performed between early and late passage at the indicated time points. Error bars represent standard deviations of technical triplicates (*n* = 3). ns, not significant.

The crypts of duodenum, jejunum, and ileum from 10-day old pig were isolated and embedded in basement membrane extract (Matrigel) and covered with porcine intestinal organoids (PIO) medium, which is modified from human intestinal organoids culture system. The organoids with a crypt-villus architecture formed in several days (Figure 1A) resembled that seen in human organoid cultures, which is already well established in our laboratory (Liu et al., 2018). Also, the structure and composition of organoids were confirmed by hematoxylin and eosin (H&E) stain (Figure 1A).

The proliferating cells, including LGR5 + cells and transit-amplifying cells within the organoid crypt domains, were confirmed by 5-ethynyl-2′-deoxyuridine (EdU) staining. The result showed that proliferating cells were present throughout the organoids under this culture condition (Figure 1B). Porcine intestinal organoids grow very robustly and rapidly and need to be passaged every 5–6 days under this culture condition. Porcine intestinal organoids were passaged by enzyme digestion at 1:4 to 1:6 ratios for at least 35 passages, which equates to approximately 6 months in culture, proliferating at comparable rates regardless of passage number (Figure 1C). Altogether, our culture conditions allow long-term expansion of porcine intestinal organoids.

### Long-Term Cultured Porcine Intestinal Organoids Retain a Differentiation Program

Above we showed that porcine intestinal organoids could proliferation for several months suggesting those long-term cultured organoids are capable of self-renewal. We then sought to determine the potential of differentiation of these long-term organoids. Wnt signaling is essential for maintaining crypt proliferation and intestinal stem cell function *in vivo* (Van De Wetering et al., 2002; Pinto et al., 2003). And the reduced Wnt signaling activity induces differentiation in mouse and human intestinal epithelial organoids. Therefore, we examined mRNA expression of differentiated cell markers in duodenum, jejunum, and ileum organoids grown in PIO medium with DAPT. DAPT is an inhibitor of the γ-secretase complex, which plays a crucial role in activating the Notch signaling. Therefore, DAPT indirectly inhibits the Notch pathway. Inhibition of the Notch pathway promotes the differentiation of mouse and human organoids (Ootani et al., 2009; Jung et al., 2011; Patel et al., 2013) and rapidly reduces the proportion of proliferating cells in rectal and ileal lines (VanDussen et al., 2015).

**FIGURE 2:**
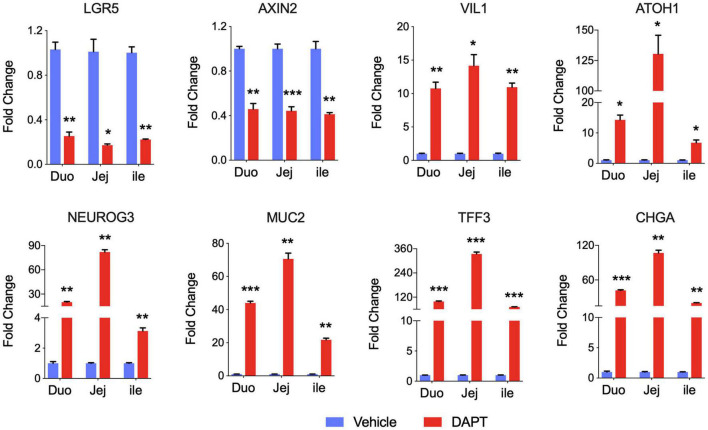
Differentiation of long-term cultured porcine intestinal organoids. qPCR gene expression analysis for LGR5, AXIN2, VIL1, ATOH1, NEUROG3, MUC2, TFF3, and CHGA. RNA was collected from the late passage of duodenum, jejunum, and ileal organoids that had been passaged for at least 30 passages, allowed to recover overnight in PIO medium, and then cultured with PIO medium with DMSO or 5% L-WRN conditioned medium with the addition of 5 mM DAPT for an additional 2 days. Data are presented as fold change relative to organoids in PIO medium. Statistics analysis were performed between DMSO and DAPT treated group (*P < 0.05, **P < 0.01, ***P < 0.001, *n* = 3, technical repeat).

As expected, intestinal stem cell marker LGR5 expression was greatly decreased, and expression of differentiated cell markers was generally increased in intestinal organoids cultured with DAPT compared with vehicle DMSO control. We also examined other stem cell marker Axin2, and the mRNA level was dramatically decreased after differentiation (Figure 2). The expression level of villin (VIL1) for enterocytes increased, demonstrating that organoid cells could differentiate into enterocytes, which are major targets of PEDV and TGEV *in vivo* (Cui et al., 2020). The addition of DAPT in the differentiation medium was important for inducing the expression of secretory cell markers (VanDussen et al., 2015), including transcription factors atonal homolog 1 (ATOH1) and neurogenin 3 (NEUROG3), which were significantly upregulated in DAPT treatment culture (Figure 2). The mRNA levels of goblet cell markers mucin 2 (MUC2) and trefoil factor 3 (TFF3) as well as endocrine cell marker chromogranin A (CHGA) increased significantly compared to undifferentiated intestinal organoids (Figure 2). These data indicated that long-term cultured porcine intestinal organoids maintained differentiation potential.

### Long-Term Cultured Intestinal Organoids Are Susceptible to Infection by Porcine Enteric Coronaviruses

**FIGURE 3:**
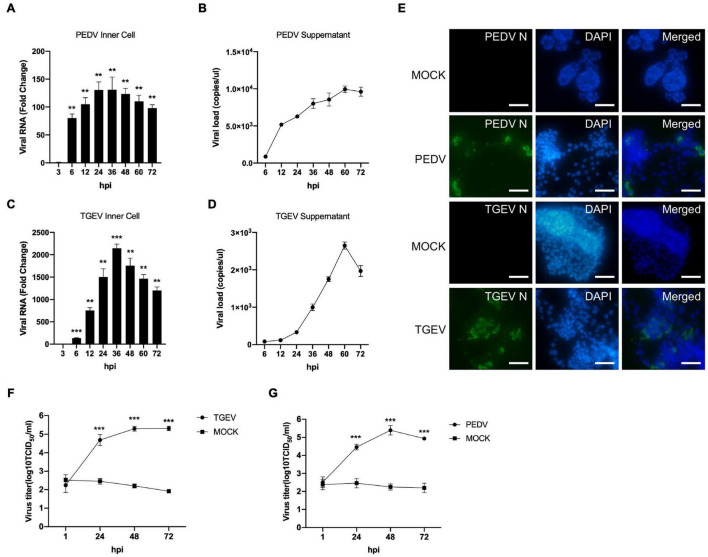
PEDV and TGEV replicate in porcine intestinal organoids. **(A–D)** PEDV or TGEV genomic levels in supernatants and inner cells of infected jejunum organoids at indicated time points after infection were quantified using an RT-PCR assay. Error bars represent standard deviations of technical triplicates. hpi, hours post-infection; **P < 0.01, ***P < 0.001, *n* = 3 per group. **(E)** TGEV-infected, PEDV-infected, or MOCK-infected jejunum organoids were collected at 48 hpi, fixed, permeabilized, and stained with TGEV N protein (green) or PEDV N protein antibody (green). DAPI was used for nuclear staining. Images were taken using a microscope. Scale bar = 200 μm. **(F,G)** The infectious PEDV or TGEV in the supernatant of organoids was titrated by TCID_50_ assay on Vero or LLC-PK1 cell line. ***P < 0.001, *n* = 3 per group.

To investigate whether long-term cultured porcine organoids could be infected by swine enteric virus, two well-characterized swine enteric coronaviruses, PEDV and TGEV, were used to infect jejunum organoids. PEDV primarily infects the small intestine and can replicate in the duodenum, jejunum, and ileum region of the small intestine *in vivo* (Li et al., 2019; Zhang et al., 2020). Previous studies showed that the load of PEDV in the jejunum was significantly higher than that in other tissues (Li et al., 2018). So we choose long-term cultured porcine jejunum organoids for further investigation. Accessing the apical surface is challenging because the apical surface of the organoids is enclosed within organoids. To make the apical surface of organoids accessible to virus challenges. First, organoids were broken up into chunks with TrypLE and then were incubated with 0.1 MOI of PEDV. Infected organoids and culture supernatant were then collected at different time points post-infection for viral replication detection by RT-qPCR. The results showed that the numbers of PEDV genomes inner cells of organoids were substantially increased after PEDV infection (Figure 3A), indicating that PEDV can infect long-term culture porcine intestinal organoids. In addition, viral load in culture supernatant remained increasing with the culture duration and peaked at 60 hpi (Figure 3B). PEDV infection in jejunum organoids 24 h post-infection was further confirmed by an anti-PEDV nucleocapsid immunofluorescence assay (IFA) (Figure 3E). In a parallel TGEV infection experiment, we inoculated porcine jejunum organoids with the TGEV at 0.1 MOI. A kinetic of viral genome replication in organoids (Figure 3C) and viral copies in the supernatant can be observed (Figure 3D), and the TGEV infection in organoids also was confirmed by IFA at 24 hpi with an anti-TGEV nucleocapsid antibody (Figure 3E). Furthermore, the infectious PEDV or TGEV in the supernatant of organoids culture was titrated by TCID_50_ assay. The results showed that infectious viral titers of the PEDV and TGEV remained increasing with the culture duration (Figures 3F,G). Collectively, these data show that long-term cultured swine intestinal organoids are permissive to swine enteric CoVs infection and are capable of supporting virus replication and progeny release.

### Transcriptome Analysis of Swine Enteric Coronaviruses Infection of Porcine Intestinal Organoids

**FIGURE 4:**
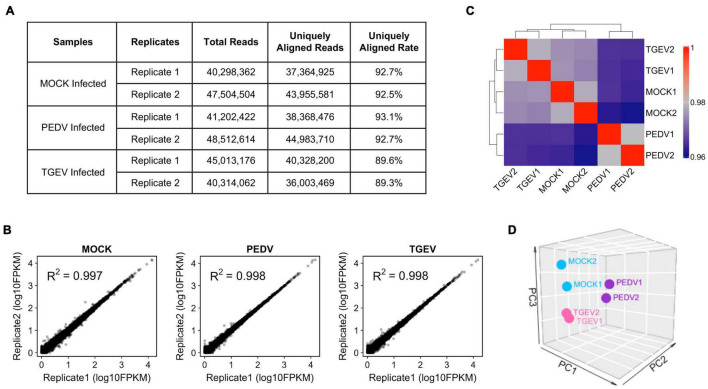
Overview of RNA-seq results from MOCK, PEDV, or TGEV infected porcine jejunum organoids. **(A)** Summary of total and uniquely aligned reads for each sample of coronavirus infected porcine jejunum organoids. **(B)** Scatterplot evaluation of the reproducibility of two different biological repeats. **(C)** Correlation heat map of MOC, PEDV, or TGEV infected organoids based on all genes expression. The colors represent pairwise Pearson correlations derived from the expression values of all genes. The hierarchical clustering is based on minus correlation distance. **(D)** PCA analysis was performed on the FPKM expression matrix of all samples.

The cells transcript landscape with enterovirus infections of the swine gastrointestinal tract remains unknown. With the goal of better understanding how porcine intestinal organoids respond to swine enteric CoVs infection, we performed RNA sequencing (RNA-seq) experiments using porcine intestinal organoids infected with 0.1 MOI of PEDV or TGEV and evaluated the transcript landscape of long-term cultured porcine jejunum organoids at 48 h after viral challenges. We obtained more than 36 million uniquely mapped reads for each sample (Figure 4A), with two biological replicates of each sample being highly reproducible (Figure 4B). Figure 4C shows the correlation heatmap of the different virus-infected organoid samples. Organoids infected with the same virus clustered tightly together. Consistent with the sample correlation analysis, we observed that RNA-seq samples from PEDV-, TGEV-, or MOCK-infected organoids could be distinguished when visualized by principal component analysis (PCA) (Figure 4D).

**FIGURE 5:**
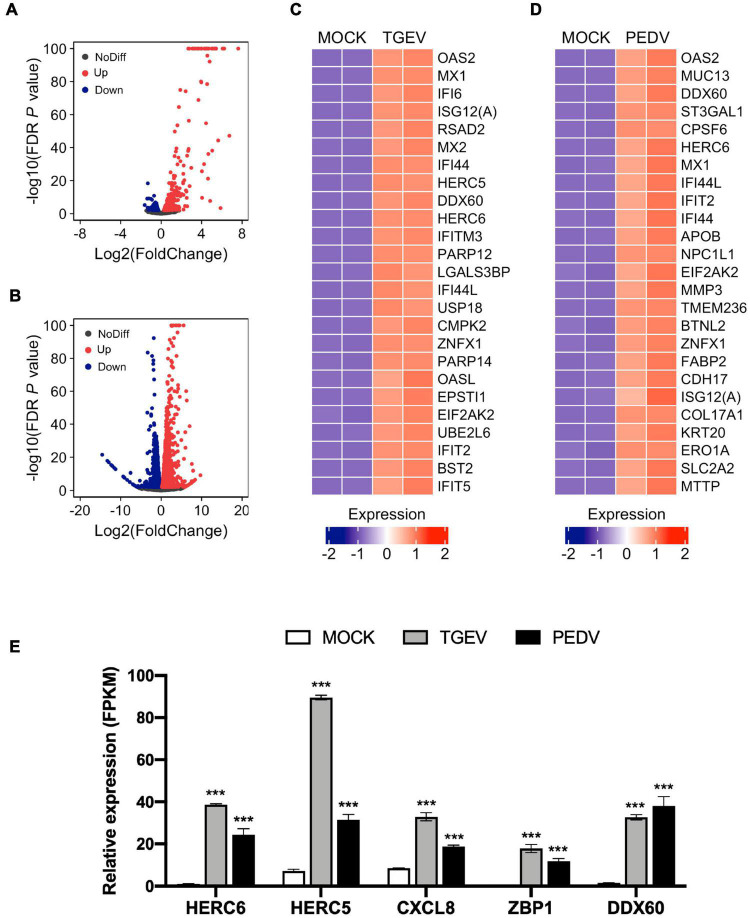
Global differential gene expression analysis of TGEV and PEDV infected organoids. **(A)** Volcano plots comparing differential gene expression levels between MOCK infected and TGEV infected organoids and **(B)** between MOCK infected and PEDV infected organoids. Red dots: up-regulated genes (FDR *P* < 0.01); Blue dots: down-regulated genes (FDR *P* < 0.01); Black dots: no difference genes. **(C)** Heatmap depicting the 25 most significantly enriched genes (FDR *P* < 0.01) upon TGEV infection in swine intestinal organoids. **(D)** Heatmap shows the top 25 enriched genes (FDR *P* < 0.01) from PEDV infected organoids. The colored bar represents the z-score of FPKM values. **(E)** RNA-seq based gene expression values (FPKM) for HERC6, HERC5, CXCL8, ZBP1, DDX60 in PEDV-, TGEV- and MOCK-infected organoids. Asterisks indicate statistically significant differences (***P < 0.001) for expression in MOCK-infected organoids relative to expression values in PEDV-infected or TGEV-infected organoids. Statistics analysis were performed between MOCK and PEDV- or TGEV-infected organoids.

Next, we examined changes in gene expression induced upon TGEV or PEDV infection compared to MOCK infection. The volcano plot of the result showed that there were 749 significantly differentially expressed genes (Supplementary Table 3), which included 485 upregulated and 264 downregulated genes in response to TGEV infection compared to that in uninfected organoids (Figure 5A). In contrast, there were 5104 significantly differentially expressed genes (Supplementary Table 4), which included 2597 upregulated and 2507 downregulated genes in response to PEDV infection compared to MOCK-infected organoids (Figure 5B). Among the highly upregulated genes in TGEV infection, most of them were interferon-stimulated genes (ISGs), including OAS2, MX1, IFI6, ISG12(A), MX2, IFITM3, USP18, OASL, IFIT2, BST2, and IFIT5 (Figure 5C). In PEDV infection, ISGs including OAS2, MX1, IFI6, IFIT2, IFI44, ISG12(A), and MX2 ranked in the top 25 of upregulated genes (Figure 5D). Other upregulated genes in both PEDV and TGEV infection, such as HERC5, HERC6, and CXCL8 (IL8), are inflammatory cytokines related to the inflammatory response, ZBP1 is Z-DNA binding protein inducing type-I interferon production, and DDX60 functions as an antiviral factor and promotes RIG-I-like receptor-mediated signaling (Figure 5E). These results showed that enteric CoVs infection could substantially change the transcript landscape of porcine intestinal organoids.

Swine enteric coronaviruses infection induce transcripts associated antiviral signaling in long-term culture organoids.

**FIGURE 6:**
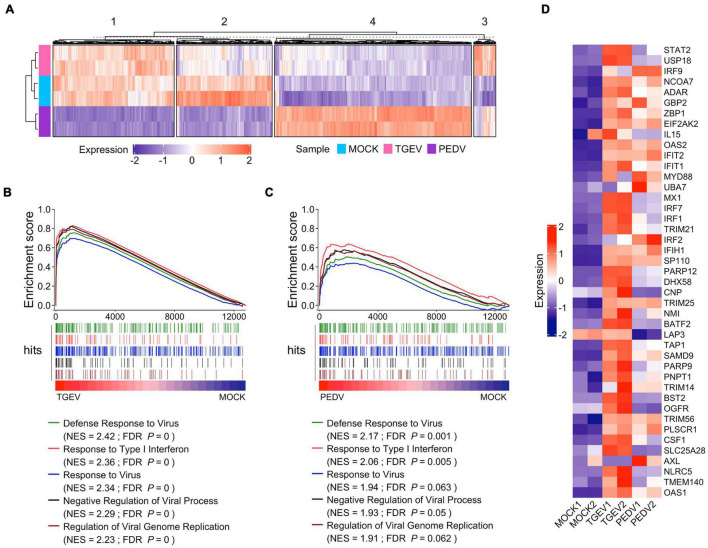
Enteric coronavirus infection induces transcripts associated with antiviral signaling in long-term culture organoids. **(A)** The unsupervised hierarchical cluster of all differentially expressed genes in PEDV and TGEV infection was divided into four groups. The colored bar represents the Z-score of the FPKM value of each gene. **(B)** GSEA plots showing top five gene signatures from GO biological process in transcriptomes of TGEV- vs. MOCK-infected organoids. **(C)** GSEA plots showing the corresponding signatures in PEDV- vs. MOCK-infected organoids, NES: normalized enrichment score; FDR *P*: False discovery rate adjusted *P* values. **(D)** Heatmaps depicting ISGs reported in Drummond et al. (2017) upon TGEV infection or PEDV infection in porcine intestinal organoids. The colored bar represents the z-score of FPKM of each gene.

We further examined the difference in gene expression between TGEV and PEDV infected organoids. Unsupervised hierarchical cluster analysis of all differentially expressed genes in TGEV and PEDV infected organoids showed that there were four groups: (1) those that are downregulated in PEDV infection (group 1); (2) those that are repressed in both TGEV and PEDV infection (group 2); (3) those that are activated in both TGEV and PEDV infection (group 3); (4) those that are highly upregulated in PEDV infection (group 4). These data suggest that PEDV and TGEV induce different transcriptomes in porcine intestinal organoids (Figure 6A).

We then performed gene sets enrichment analysis (GSEA) to evaluate the global change of porcine intestinal organoids in response to CoVs infection. Infection with TGEV elicited a broad signature associated with antiviral signaling, including cytokines and ISGs attributed to type I and III IFN responses (Figure 6B and Supplementary Table 5). PEDV also induced transcripts associated with antiviral signaling but to a much lower level than TGEV infection (Figure 6C and Supplementary Table 6), suggesting that PEDV counteracts the host’s innate immune response. PEDV develops a set of elaborate mechanisms to evade or inhibit the host antiviral innate immune response during virus evolution. These results are consistent with the innate immune suppression in PEDV infection (Li S. et al., 2020). We confirmed this by analyzing the ISGs that can be induced by human enteroviruses as well as the coronavirus infection in human intestinal organoids (Drummond et al., 2017; Lamers et al., 2020) and found that most of these ISGs were upregulated upon TGEV infection, but to fewer ISGs upregulated and much lower level in PEDV infection (Figure 6D). Altogether, these results showed that enteric coronavirus infection-induced transcripts associated with antiviral signaling in long-term cultured organoids suggest that long-term expansion organoids represent versatile models for the *in vitro* study of innate immune response to swine enteric CoVs infection.

### Porcine Epidemic Diarrhea Virus Infection Induces a Transcriptional Immunosuppression Program

**FIGURE 7:**
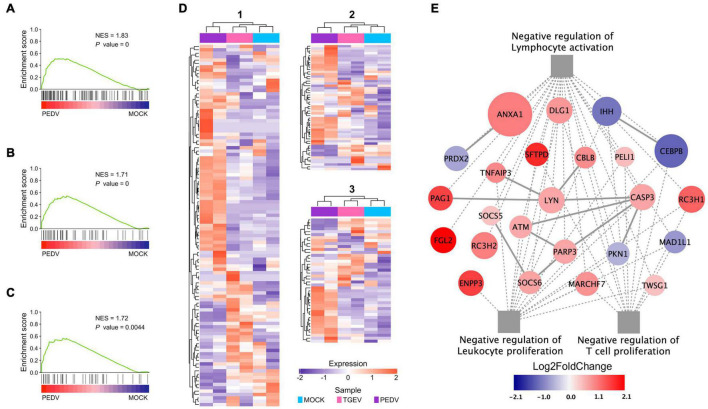
Functional analysis of PEDV on adaptive immune suppression. **(A–C)** GSEA plots show gene signatures of negative regulation of lymphocyte activation, leukocyte proliferation, and negative regulation of T cell proliferation. NES: normalized enrichment score; *P* value: FDR adjusted. **(D)** Heatmaps depicting gene expression levels of three gene signatures, 1: Negative regulation of lymphocyte activation; 2: Negative regulation of leukocyte proliferation 3: Negative regulation of T cell proliferation. The colored bar represents the z-score of FPKM of each gene. **(E)** The interaction diagram of the negative regulation of lymphocyte activation, leukocyte proliferation, and T cell proliferation. Network nodes and edges represent proteins and protein-protein associations. Gray solid lines represent protein-protein interaction from STRING Database. The gray dot lines represent the GO biological processes. The red to dark blue color bar represents the fold change of gene expression level from increasing to decreasing. The significance of the gene expression represented by –log10(*P*-value) was shown by relative diameters of a circle with the largest diameter as most significant.

In addition to inducing lower levels of ISGs and cytokines in PEDV infected organoids, our GSEA showed, interestingly, that “negative regulation of lymphocyte activation” (Figure 7A), “negative regulation of leukocyte proliferation” (Figure 7B), and “negative regulation of T cell proliferation” (Figure 7C) were identified as the top regulated processes (Supplementary Table 6). Activation-induced proliferation and clonal expansion of antigen-specific lymphocytes are a hallmark of pathogens’ adaptive immune response. Therefore, we examined genes expression levels in these three gene sets found that PEDV infection organoids present a unique expression pattern (Figure 7D), in which many genes involved in these three gene sets were dramatically upregulated, including phospholipase A2 inhibitory protein (ANXA1), RING finger and CCCH-type zinc finger domain-containing protein 1 (RC3H1), pulmonary surfactant-associated protein D (SFTPD) (Figure 7E). These results imply that the PEDV infection may induce a subset of cytokines, which affect lymphocyte activation and T cell proliferation, which in turn may damage the adaptive immune response during PEDV infection.

## Discussion

Intestinal organoids represent a significant advantage over traditional cell line models and provide a unique opportunity to explore host-pathogen interactions in an *ex vivo* system that recapitulates the complicated cellularity of the gastrointestinal tract. Swine intestinal organoids have been established with different culture systems. One previous study shows that WRN conditioned media is sufficient for *in vitro* propagation of porcine intestinal organoids (Powell and Behnke, 2017). However, WRN conditioned media only didn’t support the long-term expansion of swine intestinal organoids in our study (data not shown). Although others have generated swine intestinal organoids with commercial mouse intestinal organoids growth medium (Li et al., 2019; Li Y. et al., 2020), a study shows that porcine intestinal organoids cultured with IntestiCult™ organoid growth medium for mouse intestinal organoids seem as such less useful to study the interaction of enterotoxins or pathogens with the intestinal epithelium (Vermeire et al., 2021). In addition, the mouse organoid growth medium may not allow long-term expansion of swine intestinal organoids. Here, we describe a versatile approach by modifying previously defined insights in the growth requirements of the intestinal epithelium of humans (Sato et al., 2011; Liu et al., 2018) to establish swine intestinal epithelial organoids. Major differences to other swine intestinal organoid models are their long-term piglet expansion. Studies of these cultures indicate that those swine intestinal organoids can self-renew in culture for 6 months at least. The differentiation experiment of these long-term cultured organoids also indicated that the organoids under this culture condition maintained differentiation potential.

A challenge in using organoids to study virus-host interactions is that the apical surface is enclosed within the organoids, and therefore it is difficult to access the virus, which hampers the application of intestinal organoids in virus-host interaction studies. Several techniques, including microinjection, 3D to 2D culture, and apical-out organoid culture, were developed (Co et al., 2019; Li Y. et al., 2020). However, the procedures of these methods were relatively complicated. This study used TrypLE to break intestinal organoids into large pieces and incubated them with swine enteric coronaviruses. The virus infection experiment indicated that this technique was efficient for the virus to access the apical surface of organoids. This approach for organoid infection was also reported to be used for CoVID-19 infection of human intestinal organoids (Lamers et al., 2020).

Subsequently, two swine enteric coronaviruses, PEDV and TGEV, were employed to verify the infectivity of long-term cultured porcine intestinal organoids. We exposed long-term cultured porcine jejunum organoids to TGEV and PEDV at an MOI of 0.1. Samples were harvested at multiple time points after infection and processed for the viral load analyses. Culture supernatants contained lower infectious virus levels than inside organoids, implying that the Matrigel may impede virus release. The limited virus in supernatant would be overcome by using a bioreactor and organoids-on-a-chip (Meran et al., 2020; Nikolaev et al., 2020), in which organoids were cultured without basement membrane extract.

Previous studies have not investigated the transcriptome change of porcine intestinal organoids upon enteric viral infection (Li et al., 2019; Li Y. et al., 2020). Here, we evaluated the transcriptional landscape of organoids upon swine enteric virus infection by RNA sequencing. Cells typically respond to virus infection by mounting an innate antiviral response to limit the spread of the infection and aid in inducing an adaptive immune response that will eventually clear the virus. The current study detected the robust induction of transcripts associated with antiviral signaling in response to swine enteric coronavirus infection of intestinal organoids, including many ISGs, cytokines, and chemokines (Supplementary Tables 3, 4). Upon infection, the small intestinal epithelium secretes pro-inflammatory mediators, such as IL8 (CXCL8) (Vermeire et al., 2021). Our data show similar swine enteric CoVs infection responses in the long-term cultured organoids. The RNA-seq result showed an upregulated IL8 expression when exposed to CoVs. IL8 functions as a chemotactic factor that attracts neutrophils, basophils, and T-cells during the inflammatory process. PEDV has evolved strategies to suppress and/or evade these immune responses, which can dramatically influence the course of the infection, including pathogenesis and persistence in the host (Li S. et al., 2020). In agreement with the previous results, we showed that PEDV could compromise the innate immune response in long-term cultured organoids. Moreover, our GSEA results implied that PEDV might damage the adaptive immune response during PEDV infection through negative lymphocyte activation and/or proliferation regulation.

In summary, we designed a robust long-term culture system to expand the porcine intestinal organoids. These intestinal organoids were susceptible to infection by porcine enteric coronavirus PEDV and TGEV. Our findings provide important insights into events associated with PEDV and TGEV infection and demonstrate that swine intestinal organoids can be used as an *in vitro* model to define the complicated cross-talk between enteric coronavirus and the intestinal epithelium. This 3D intestinal organoid model offers a long-term, renewable resource for investigating porcine intestinal infections with various pathogens.

## Data Availability Statement

The datasets presented in this study can be found in online repositories. The names of the repository/repositories and accession number(s) can be found in the article/Supplementary Material.

## Ethics Statement

The animal study was reviewed and approved by the Ethical Committee of the Shanghai Veterinary Research Institute, Chinese Academy of Agricultural Sciences.

## Author Contributions

CL and GT: conceptualization, supervision, and funding acquisition. MZ, LLv, HC, WT, and CL: methodology. CL, MZ, and LY: software. YJ, HC, and LY: validation. CL, MZ, and LLv: formal analysis and data curation. MZ, LLv, and HC: investigation. FG, GL, WT, YL, and LLi: resources. CL: writing—original draft preparation. CL, GT, LY, and FG: writing—review and editing. YJ and CL: visualization. CL and FG: project administration. All authors have read and agreed to the published version of the manuscript.

## Conflict of Interest

The authors declare that the research was conducted in the absence of any commercial or financial relationships that could be construed as a potential conflict of interest. The reviewer XL declared a shared affiliation with the authors YL, FG, LY, YJ, WT, LLi, GL, GT, and CL to the handling editor at the time of the review.

## Publisher’s Note

All claims expressed in this article are solely those of the authors and do not necessarily represent those of their affiliated organizations, or those of the publisher, the editors and the reviewers. Any product that may be evaluated in this article, or claim that may be made by its manufacturer, is not guaranteed or endorsed by the publisher.

## Footnotes

1
http://asia.ensembl.org/Sus_scrofa/Info/Annotation

2
https://www.r-project.org/

3
https://www.gsea-msigdb.org/gsea/index.jsp

4
https://www.gsea-msigdb.org/gsea/msigdb/collections.jsp#C5

## References

[B1] Artegiani B. Clevers H. (2018). Use and application of 3D-organoid technology. *Hum. Mol. Genet.* 27 R99–R107. 10.1093/hmg/ddy187 29796608

[B2] Clevers H. (2016). Modeling development and disease with organoids. *Cell* 165 1586–1597. 10.1016/j.cell.2016.05.082 27315476

[B3] Co J. Y. Margalef-Catala M. Li X. Mah A. T. Kuo C. J. Monack D. M. (2019). Controlling epithelial polarity: a human enteroid model for host-pathogen interactions. *Cell Rep.* 26 2509.e2504–2520.e2504. 10.1016/j.celrep.2019.01.108 30811997 PMC6391775

[B4] Cui T. Theuns S. Xie J. Van Den Broeck W. Nauwynck H. J. (2020). Role of porcine aminopeptidase N and sialic Acids in porcine coronavirus infections in primary porcine enterocytes. *Viruses* 12:402. 10.3390/v12040402 32260595 PMC7232180

[B5] Derricott H. Luu L. Fong W. Y. Hartley C. S. Johnston L. J. Armstrong S. D. (2019). Developing a 3D intestinal epithelium model for livestock species. *Cell Tissue Res.* 375 409–424. 10.1007/s00441-018-2924-9 30259138 PMC6373265

[B6] Drummond C. G. Bolock A. M. Ma C. Luke C. J. Good M. Coyne C. B. (2017). Enteroviruses infect human enteroids and induce antiviral signaling in a cell lineage-specific manner. *Proc. Natl. Acad. Sci. U.S.A.* 114 1672–1677. 10.1073/pnas.1617363114 28137842 PMC5320971

[B7] Fujii M. Matano M. Nanki K. Sato T. (2015). Efficient genetic engineering of human intestinal organoids using electroporation. *Nat. Protoc.* 10 1474–1485. 26334867 10.1038/nprot.2015.088

[B8] Gu Z. Eils R. Schlesner M. (2016). Complex heatmaps reveal patterns and correlations in multidimensional genomic data. *Bioinformatics* 32 2847–2849. 10.1093/bioinformatics/btw313 27207943

[B9] Hofmann M. Wyler R. (1988). Propagation of the virus of porcine epidemic diarrhea in cell culture. *J. Clin. Microbiol.* 26 2235–2239. 10.1128/jcm.26.11.2235-2239.1988 2853174 PMC266866

[B10] Jung K. Hu H. Saif L. J. (2016). Porcine deltacoronavirus infection: etiology, cell culture for virus isolation and propagation, molecular epidemiology and pathogenesis. *Virus Res.* 226 50–59. 10.1016/j.virusres.2016.04.009 27086031 PMC7114557

[B11] Jung K. Saif L. J. (2015). Porcine epidemic diarrhea virus infection: etiology, epidemiology, pathogenesis and immunoprophylaxis. *Vet. J.* 204 134–143. 10.1016/j.tvjl.2015.02.017 25841898 PMC7110711

[B12] Jung P. Sato T. Merlos-Suarez A. Barriga F. M. Iglesias M. Rossell D. (2011). Isolation and in vitro expansion of human colonic stem cells. *Nat. Med.* 17 1225–1227. 10.1038/nm.2470 21892181

[B13] Lamers M. M. Beumer J. Van Der Vaart J. Knoops K. Puschhof J. Breugem T. I. (2020). SARS-CoV-2 productively infects human gut enterocytes. *Science* 369 50–54. 10.1126/science.abc1669 32358202 PMC7199907

[B14] Li L. Fu F. Guo S. Wang H. He X. Xue M. (2019). Porcine intestinal enteroids: a new model for studying enteric coronavirus porcine epidemic diarrhea virus infection and the host innate response. *J. Virol.* 93 e1682–e1618. 10.1128/JVI.01682-18 30541861 PMC6384061

[B15] Li S. Yang J. Zhu Z. Zheng H. (2020). Porcine epidemic diarrhea virus and the host innate immune response. *Pathogens* 9:367. 10.3390/pathogens9050367 32403318 PMC7281546

[B16] Li Y. Yang N. Chen J. Huang X. Zhang N. Yang S. (2020). Next-generation porcine intestinal organoids: an apical-out organoid model for swine enteric virus infection and immune response investigations. *J. Virol.* 94 e1006–e1020. 10.1128/JVI.01006-20 32796075 PMC7565635

[B17] Li Y. Wu Q. Huang L. Yuan C. Wang J. Yang Q. (2018). An alternative pathway of enteric PEDV dissemination from nasal cavity to intestinal mucosa in swine. *Nat. Commun.* 9:3811. 10.1038/s41467-018-06056-w 30232333 PMC6145876

[B18] Liu C. Banister C. E. Weige C. C. Altomare D. Richardson J. H. Contreras C. M. (2018). PRDM1 silences stem cell-related genes and inhibits proliferation of human colon tumor organoids. *Proc. Natl. Acad. Sci. U.S.A.* 115 E5066–E5075. 10.1073/pnas.1802902115 29760071 PMC5984534

[B19] Liu C. Tang J. Ma Y. Liang X. Yang Y. Peng G. (2015). Receptor usage and cell entry of porcine epidemic diarrhea coronavirus. *J. Virol.* 89 6121–6125. 10.1128/JVI.00430-15 25787280 PMC4442452

[B20] Love M. I. Huber W. Anders S. (2014). Moderated estimation of fold change and dispersion for RNA-seq data with DESeq2. *Genome Biol.* 15:550. 10.1186/s13059-014-0550-8 25516281 PMC4302049

[B21] Meran L. Massie I. Campinoti S. Weston A. E. Gaifulina R. Tullie L. (2020). Engineering transplantable jejunal mucosal grafts using patient-derived organoids from children with intestinal failure. *Nat. Med.* 26 1593–1601. 10.1038/s41591-020-1024-z 32895569 PMC7116539

[B22] Miyoshi H. Stappenbeck T. S. (2013). In vitro expansion and genetic modification of gastrointestinal stem cells in spheroid culture. *Nat. Protoc.* 8 2471–2482. 10.1038/nprot.2013.153 24232249 PMC3969856

[B23] Nikolaev M. Mitrofanova O. Broguiere N. Geraldo S. Dutta D. Tabata Y. (2020). Homeostatic mini-intestines through scaffold-guided organoid morphogenesis. *Nature* 585 574–578. 10.1038/s41586-020-2724-8 32939089

[B24] Ootani A. Li X. Sangiorgi E. Ho Q. T. Ueno H. Toda S. (2009). Sustained in vitro intestinal epithelial culture within a Wnt-dependent stem cell niche. *Nat. Med.* 15 701–706. 10.1038/nm.1951 19398967 PMC2919216

[B25] Patel K. K. Miyoshi H. Beatty W. L. Head R. D. Malvin N. P. Cadwell K. (2013). Autophagy proteins control goblet cell function by potentiating reactive oxygen species production. *EMBO J.* 32 3130–3144. 10.1038/emboj.2013.233 24185898 PMC3981139

[B26] Pinto D. Gregorieff A. Begthel H. Clevers H. (2003). Canonical Wnt signals are essential for homeostasis of the intestinal epithelium. *Genes Dev.* 17 1709–1713. 10.1101/gad.267103 12865297 PMC196179

[B27] Powell R. H. Behnke M. S. (2017). WRN conditioned media is sufficient for in vitro propagation of intestinal organoids from large farm and small companion animals. *Biol. Open* 6 698–705. 10.1242/bio.021717 28347989 PMC5450310

[B28] Sato T. Stange D. E. Ferrante M. Vries R. G. Van Es J. H. Van Den Brink S. (2011). Long-term expansion of epithelial organoids from human colon, adenoma, adenocarcinoma, and Barrett’s epithelium. *Gastroenterology* 141 1762–1772. 21889923 10.1053/j.gastro.2011.07.050

[B29] Sato T. Vries R. G. Snippert H. J. Van De Wetering M. Barker N. Stange D. E. (2009). Single Lgr5 stem cells build crypt-villus structures in vitro without a mesenchymal niche. *Nature* 459 262–265. 10.1038/nature07935 19329995

[B30] Subramanian A. Tamayo P. Mootha V. K. Mukherjee S. Ebert B. L. Gillette M. A. (2005). Gene set enrichment analysis: a knowledge-based approach for interpreting genome-wide expression profiles. *Proc. Natl. Acad. Sci. U.S.A.* 102 15545–15550. 10.1073/pnas.0506580102 16199517 PMC1239896

[B31] Sungsuwan S. Jongkaewwattana A. Jaru-Ampornpan P. (2020). Nucleocapsid proteins from other swine enteric coronaviruses differentially modulate PEDV replication. *Virology* 540 45–56. 10.1016/j.virol.2019.11.007 31756532 PMC7112109

[B32] Van De Wetering M. Sancho E. Verweij C. De Lau W. Oving I. Hurlstone A. (2002). The beta-catenin/TCF-4 complex imposes a crypt progenitor phenotype on colorectal cancer cells. *Cell* 111 241–250. 10.1016/s0092-8674(02)01014-0 12408868

[B33] VanDussen K. L. Marinshaw J. M. Shaikh N. Miyoshi H. Moon C. Tarr P. I. (2015). Development of an enhanced human gastrointestinal epithelial culture system to facilitate patient-based assays. *Gut* 64 911–920. 10.1136/gutjnl-2013-306651 25007816 PMC4305344

[B34] Vermeire B. Gonzalez L. M. Jansens R. J. J. Cox E. Devriendt B. (2021). Porcine small intestinal organoids as a model to explore ETEC-host interactions in the gut. *Vet. Res.* 52:94. 34174960 10.1186/s13567-021-00961-7 PMC8235647

[B35] Vlasova A. N. Wang Q. Jung K. Langel S. N. Malik Y. S. Saif L. J. (2020). “Porcine coronaviruses,” in *Emerging and Transboundary Animal Viruses*, eds Malik Y. Singh R. Yadav M. (Singapore: Springer), 79–110.

[B36] Wang X. Fang L. Liu S. Ke W. Wang D. Peng G. (2019). Susceptibility of porcine IPI-2I intestinal epithelial cells to infection with swine enteric coronaviruses. *Vet Microbiol* 233 21–27. 10.1016/j.vetmic.2019.04.014 31176408 PMC7117161

[B37] Zhang Q. Ma J. Yoo D. (2017). Inhibition of NF-kappaB activity by the porcine epidemic diarrhea virus nonstructural protein 1 for innate immune evasion. *Virology* 510 111–126. 10.1016/j.virol.2017.07.009 28715653 PMC7111422

[B38] Zhang Q. Shi K. Yoo D. (2016). Suppression of type I interferon production by porcine epidemic diarrhea virus and degradation of CREB-binding protein by nsp1. *Virology* 489 252–268. 10.1016/j.virol.2015.12.010 26773386 PMC7111358

[B39] Zhang S. Cao Y. Yang Q. (2020). Transferrin receptor 1 levels at the cell surface influence the susceptibility of newborn piglets to PEDV infection. *PLoS Pathog* 16:e1008682. 10.1371/journal.ppat.1008682 32730327 PMC7419007

[B40] Zhao S. Gao J. Zhu L. Yang Q. (2014). Transmissible gastroenteritis virus and porcine epidemic diarrhoea virus infection induces dramatic changes in the tight junctions and microfilaments of polarized IPEC-J2 cells. *Virus Res.* 192 34–45. 10.1016/j.virusres.2014.08.014 25173696 PMC7114495

