# Supplementary material for: Long-Term Expansion of Porcine Intestinal Organoids Serves as an in vitro Model for Swine Enteric Coronavirus Infection
Source: Front Microbiol. 2022 Mar 14;13:865336. doi: 10.3389/fmicb.2022.865336 (PMC8967161; doi:10.3389/fmicb.2022.865336)
Supplement: Supplementary file 1 [file Table_1.DOCX]

Table S1. Reagents information of Porcine Intestinal Organoids Medium

| **Reagent Name** | **Catalog Number** | **Supplier** |
| --- | --- | --- |
| Advanced DMEM/F12 | 12634-010 | Invitrogen |
| Primocin | ant-pm-1 | Invivogen |
| GlutaMAX™ Supplement | 35050-061 | Invitrogen |
| HEPES (1 M) | 15630-080 | Invitrogen |
| N-2 Supplement (100X) | 17502-048 | Invitrogen |
| B-27® Supplement (50X), minus vitamin A | 12587-010 | Invitrogen |
| N-Acetyl-L-cysteine | A9165-5G | Sigma-Aldrich |
| EGF | PHG0311 | Invitrogen |
| A-83-01 | SML0788-5mg | Sigma |
| SB202190 | S7067-5MG | Sigma-Aldrich |
| Nicotinamide | N0636-100G | Sigma-Aldrich |
