# Supplementary material for: Long-Term Expansion of Porcine Intestinal Organoids Serves as an in vitro Model for Swine Enteric Coronavirus Infection
Source: Front Microbiol. 2022 Mar 14;13:865336. doi: 10.3389/fmicb.2022.865336 (PMC8967161; doi:10.3389/fmicb.2022.865336)
Supplement: Supplementary file 2 [file Table_2.DOCX]

Table S2 Primers used in organoids differentiation experiment

| **Primer name** | **Sequence (5’-3’)** | **Reference No.** |
| --- | --- | --- |
| Porcine-qPCR-GAPDH-F | GGTGCTACGTATGTTGTGGA | NM_001206359.1 |
| Porcine-qPCR-GAPDH-R | CTGACGATCTTGAGGGAGTT |  |
| Porcine-qPCR-ATOH1-F | GGGCTGAAGTGAAGGAGTTG | XM_003129319.4 |
| Porcine-qPCR-ATOH1-R | CCCAGCTCTGGAGAATGAAG |  |
| Porcine-qPCR-AXIN2-F | GAGGGAGAAATGCGTGGATA | XM_021066736 |
| Porcine-qPCR-AXIN2-R | GGTTTCAGCTGCTTGGAGAC |  |
| Porcine-qPCR-CHGA-F | TCGAGGTCATCTCTGACACG | NM_001164005.2 |
| Porcine-qPCR-CHGA-R | TTCTTCTGCTGATGGGACCT |  |
| Porcine-qPCR-LGR5-F | AATTCCCTTTGCTTCCTGGT | NM_001315762.1 |
| Porcine-qPCR-LGR5-R | GGGCTGATGAATGTGAGGTT |  |
| Porcine-qPCR-MUC2-F | AACTGCGAGCAATGTGTCTG | XM_021082584.1 |
| Porcine-qPCR-MUC2-R | CAGGTCTGCTTGTCTGTGGA |  |
| Porcine-qPCR-NEUROG3-F | TGAAGTCTGTCTGCCCCTCT | XM_021072424.1 |
| Porcine-qPCR-NEUROG3-R | GGCTCTGAAAAGTGCAGGAG |  |
| Porcine-qPCR-TFF3-F | CCAGGGCCTGATGTCTTAAA | NM_001243483.1 |
| Porcine-qPCR-TFF3-R | AAAGTGGCACCAAGAAGTGG |  |
| Porcine-qPCR-VIL1-F | CTACCCTGCCTGCAGACTTC | XM_001925167.6 |
| Porcine-qPCR-VIL1-R | GCAACCACCTTCTTGAGAGC |  |
